# First-in-Human Imaging Results of PHAROS: A Versatile High-Resolution TOF/DOI PET Scanner for Brain, Breast, and Extremity Imaging

**DOI:** 10.2967/jnumed.125.271172

**Published:** 2026-08

**Authors:** Guen Bae Ko, Kyeong Yun Kim, Seung Kwan Kang, Jaehun Lee, Raehun Jung, Dong Jin Kwak, Jeong-Whan Son, Seong A. Shin, Hyun Gee Ryoo, Yeon-Koo Kang, SunGyu Kim, Jin Chul Paeng, Min Soo Byun, Jung Hwan Shin, Gi Jeong Cheon, Yu Kyeong Kim, Hongyoon Choi, Jae Sung Lee

**Affiliations:** 1 Brightonix Imaging Inc., Seoul, Korea;; 2 Institute of Radiation Medicine, Medical Research Center, Seoul National University College of Medicine, Seoul, Korea;; 3 Department of Nuclear & Quantum Engineering, Korea Advanced Institute of Science and Technology, Daejeon, Korea;; 4 Department of Nuclear Medicine, Seoul National University Hospital, Seoul, Korea;; 5 Department of Nuclear Medicine, Seoul National University College of Medicine, Seoul, Korea;; 6 Department of Neuropsychiatry, Seoul National University College of Medicine, Seoul, Korea;; 7 Department of Neurology, Seoul National University College of Medicine, Seoul, Korea; and; 8 Department of Nuclear Medicine, Seoul Metropolitan Government Seoul National University Boramae Medical Center, Seoul, Korea

**Keywords:** high-resolution PET, time of flight, depth of interaction, neuroimaging, breast cancer imaging

## Abstract

We present the design and performance of the newly developed PHAROS—a high-resolution, multifunctional PET system integrating time-of-flight (TOF) and depth-of-interaction (DOI) technologies—and report its first-in-human imaging results for brain and breast applications in multiple patient positions. **Methods:** The PHAROS system features a movable detector head, transformable patient table, and compact footprint, enabling both seated and supine neuroimaging as well as breast and extremity imaging. The scanner’s PET ring comprises 20 detector sectors, each with 3 × 12 block detectors in the evaluated 3-detector-module-ring configuration, providing a 19.6-cm axial field of view. Each block detector consists of 8 × 8 lutetium oxyorthosilicate crystals (1.92 × 1.92 × 15 mm^3^) coupled to dual 4 × 4 silicon photomultiplier arrays for DOI readout. System performance was assessed using a 3-dimensional Hoffman brain phantom and first-in-human studies, including [^18^F]FDG brain imaging, [^18^F]florbetaben amyloid PET, [^18^F]FP-CIT dopamine transporter PET, and prone-position [^18^F]FDG breast imaging. All images were reconstructed with 3-dimensional ordered-subset expectation maximization incorporating TOF and DOI information. **Results:** In the Hoffman phantom, cortical and subcortical structures were sharply visualized without artifacts, and layered activity patterns were clearly resolved in coronal and sagittal planes. In the [^18^F]FDG brain PET of a healthy volunteer, PHAROS produced high-quality images with well-defined cortical and subcortical uptake patterns and clear gray–white matter contrast. In [^18^F]florbetaben amyloid PET, the amyloid-negative case showed marked gray–white matter contrast and a characteristic spiculated pattern of white matter tracts, whereas the amyloid-positive case exhibited diffuse cortical binding of radiotracer with the relative sparing of the precentral, postcentral, and occipital cortices. Dopamine transporter PET showed reduced bilateral putaminal binding in a patient with Parkinson disease. In prone breast PET, multiple hypermetabolic masses and skin invasion were clearly delineated with high tumor-to-background contrast, also enabling detailed visualization of tumor heterogeneity. **Conclusion:** These first-in-human results demonstrate that the PHAROS system achieves remarkable spatial resolution and high image quality across diverse applications. Its versatile design, incorporating DOI and TOF technologies, supports a wide range of clinical and research uses, enabling accurate lesion characterization in both neurologic and oncologic settings.

## Visual Abstract

**Figure:**
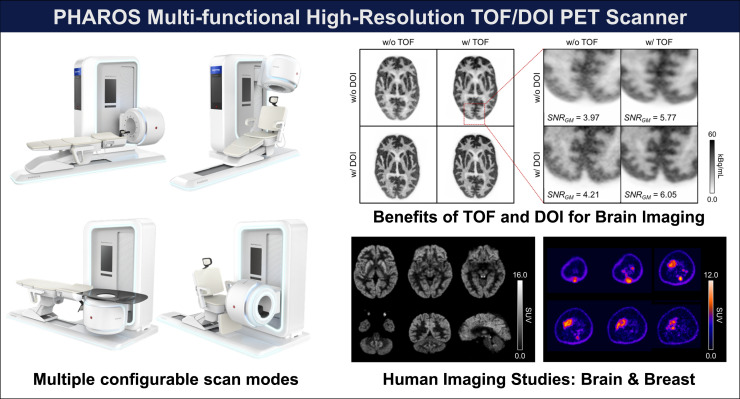

PET is a useful clinical tool for accurately visualizing the presence of biomarkers in the brain associated with various neurodegenerative diseases such as Alzheimer and Parkinson disease (e.g., amyloid-β plaques or microtubule-associated protein tau strands) (1–3). As the prevalence of neurodegenerative diseases continues to rise and new antiamyloid β-therapies emerge (4,5), the demand for brain PET scans is also increasing.

Brain-dedicated PET scanners have been developed to achieve higher image quality and spatial resolution compared with conventional whole-body PET systems (6–9). Their dedicated geometry and optimized detector configurations allow superior gray–white matter contrast, better delineation of cortical and subcortical structures, and more accurate quantification of small lesions. These advantages are particularly important in the evaluation of neurodegenerative diseases, where early detection of subtle metabolic or molecular abnormalities is critical for diagnosis and treatment monitoring. Furthermore, high-resolution dedicated brain PET systems can reduce partial-volume effects and improve sensitivity, ultimately enhancing diagnostic confidence in both clinical and research settings.

Brain PET scans can be performed in either sitting or lying position (10). Sitting position requires less installation space and may reduce patient anxiety by offering a more open environment. It is also advantageous for studies involving cognitive tasks or responses to external stimuli, as it more closely resembles natural daily activities. The lying position helps to minimize patient motion, which is critical for acquiring high-quality images.

Whole-body PET is widely used for various purposes in cancer staging and detecting metastases including breast cancer (11,12); however, standard whole-body PET scanners have limitations in accurately and quantitatively characterizing primary breast tumors. Detailed molecular characterization of breast primary tumors, including intratumoral heterogeneity, is hindered in conventional whole-body PET because of breast compression in the supine position, limited spatial resolution, and respiratory motion (13). In contrast, high-resolution dedicated breast PET scanners offer superior capability of detecting small lesions and assessing intratumoral heterogeneity (14,15). Furthermore, fully 3-dimensional breast PET imaging performed in the prone position provides better spatial correlation with contrast-enhanced MRI (16). Beyond breast cancer, there are other clinical domains where dedicated, small-gantry PET systems can offer significant added value. High-resolution PET imaging of extremities has clinical relevance for vascular disorders, such as peripheral arterial disease, and for musculoskeletal or rheumatologic conditions where subtle, localized metabolic changes must be captured (17,18).

PHAROS (Brightonix Imaging Inc.) is a newly developed, multifunctional, high-resolution PET scanner designed for supporting various patient positions. It features a flexibly movable PET detector head and patient couch, enabling a wide range of scan modes, including seated and supine neuroimaging, as well as breast and extremity imaging, all while maintaining high spatial resolution. The incorporation of time-of-flight (TOF) and depth-of-interaction (DOI) technologies further enhances image quality and ensures spatial resolution uniformity. This paper presents the design concept of the PHAROS system and demonstrates its feasibility through initial human imaging studies.

## MATERIALS AND METHODS

### System Design

PHAROS is designed to acquire high-resolution PET images of the brain, breast, and extremities in various patient postures. To enable this versatility, the system incorporates a movable detector head and a transformable patient couch. This configuration maximizes scanner use within a compact footprint, especially when compared with conventional whole-body PET/CT or PET/MRI systems.

**FIGURE 1.**
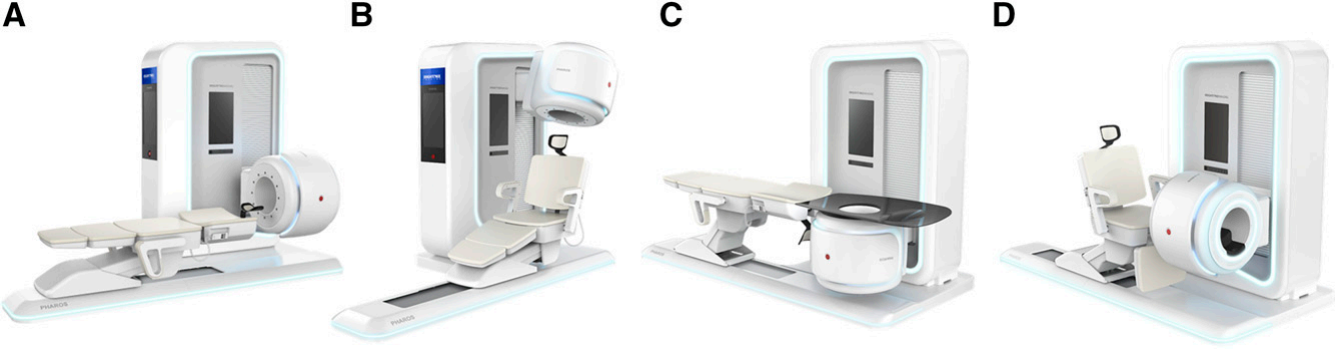
Overview of PHAROS and mechanical configuration, showing its compact design, movable detector head, and transformable patient table capable of both chair and bed configurations. (A) Brain scan mode in lying (supine) position. (B) Brain scan mode in sitting position. (C) Breast scan mode in prone position. (D) Extremity scan mode.

Mechanical movements are fully automated through 10 precision-controlled drive mechanisms, with integrated collision detection and emergency stop functions to ensure patient safety. The detector head is capable of 180° rotation, and the patient couch can be transformed between a chair and a bed configuration (Fig. 1). The detector head position can be finely adjusted using a remote controller to align the patient within the field of view (FOV), whereas a real-time projection image is displayed on the side display panel of the gantry tower to guide and confirm proper positioning. The backrest height is adjustable to accommodate patients of different body sizes. Additionally, to minimize γ-ray attenuation, the headrest is constructed from carbon fiber–reinforced plastic.

**FIGURE 2.**
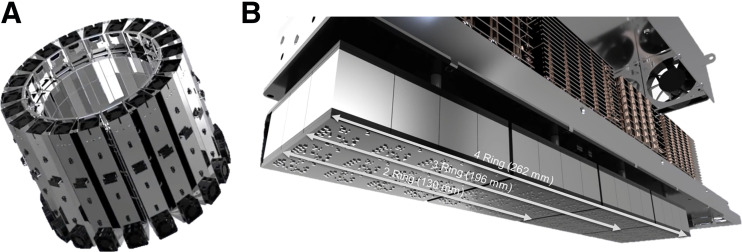
Detector ring structure and DOI-enabled detector design. (A) PET detector ring composed of 20 detector sectors with bore opening of 32 cm. (B) Detector sector.

Depending on the configuration, the PHAROS supports 3 different axial FOVs: 13.0 cm for the 2-detector module ring, 19.6 cm for the 3-detector module ring, and 26.2 cm for the 4-detector module ring (Fig. 2). In this study, we used the 3-detector-module-ring PHAROS system. The PET specifications of the PHAROS are summarized in Table 1.

**TABLE 1.** Specifications of PHAROS

Characteristic	Value
Bore opening (cm)	32
Scintillator/photosensor	LSO/SiPM
Crystal size (mm^3^)	1.92 × 1.92 × 15.0
Crystal pitch (mm)	2.0
No. of detector sectors	20
No. of crystals per ring	480
Axial FOV (cm)*	13.0 (2-detector module ring)/ 19.6 (3-detector module ring)/ 26.2 (4-detector module ring)

* Axial FOV depends on model.

LSO = lutetium oxyorthosilicate.

To simultaneously achieve precise DOI and excellent timing performance, PHAROS adopts a dual-ended readout, in which two 4 × 4 silicon photomultiplier (SiPM) arrays are coupled to the top and bottom surfaces of each crystal array (19,20). The DOI position is determined on the basis of the difference of the pulse integrals from the two SiPMs. Trigger signals from each SiPM provide 2 time stamps, which are used to estimate the accurate arrival times of the annihilation photons (21,22).

The 3-detector-module-ring PHAROS system demonstrated an average energy resolution of 11.6 ± 1.2% at 511 keV, a timing resolution of 249 ps, and a sensitivity of 8.79 cps/kBq. Near the center of the FOV, the system achieved sub–1.5-mm spatial resolution. Detailed descriptions of the system design and performance are provided in the supplemental methods (supplemental materials are available at http://jnm.snmjournals.org).

### Data Acquisition and Image Reconstruction

All data in this study were acquired in list-mode and reconstructed using line-of-response–based 3-dimensional (3D) ordered-subset expectation maximization algorithm (26 subsets and 4 iterations) with corrections for attenuation, scatter, randoms, and normalization. The reconstruction incorporated both DOI and TOF information, producing images with 1-mm isotropic voxel size and an image matrix of 281 × 281 × 196, unless otherwise specified. Axial compression of lines of response was applied with a maximum ring difference of 95 and a span of 13, covering the full axial acceptance angle. The reconstruction algorithm was accelerated using a graphics processing unit. Point-spread function modeling and motion correction were not applied in this study.

### Hoffman Phantom Study

To evaluate the spatial resolution of the scanner, a 3D Hoffman brain phantom filled with 54 MBq of [^18^F]FDG was scanned for 5 h. Attenuation and scatter corrections based on CT images acquired using a Siemens mCT PET/CT scanner were applied. To evaluate the contrast recovery between gray and white matter of the Hoffman phantom, the reconstructed images were analyzed using a method similar to that described by Omidvari et al. (23). Gray and white matter were segmented on a 4-mm-thick slice centered on each phantom layer, and the mean activity concentrations from these segmented regions were used to calculate the gray-to-white matter contrast.

Furthermore, to assess the benefits of DOI and TOF information on image quality, Hoffman phantom images were compared under 4 different reconstruction configurations while keeping all other parameters identical: without DOI and without TOF, without DOI but with TOF, with DOI but without TOF, and with both DOI and TOF. For quantitative analysis, the signal-to-noise ratio of gray matter region (SNR_GM_) was calculated as follows:

$$\mathrm{SN}R_{\mathrm{GM}}=\frac{\mu_{\mathrm{GM}}}{\sigma_{\mathrm{GM}}},$$

where μ_GM_ and σ_GM_ represent the mean and SD of voxel intensities in the segmented gray matter volume, respectively.

### Human Studies

Initial human brain PET scans were performed in the supine position.

A 51-y-old cognitively normal male volunteer underwent a 30-min brain PET scan beginning 40 min after intravenous injection of 333 MBq of [^18^F]FDG. The blood glucose level measured before tracer injection was 85 mg/dL. The list-mode dataset was binned into histograms with scan durations of 10, 20, and 30 min to assess the noise levels at different scan durations. Images were reconstructed using 3D ordered-subset expectation maximization with template-based attenuation correction, in which an initial PET image reconstructed without attenuation and scatter correction was used as the registration target, and a predefined PET template was affinely registered to this image. The resulting transformation was then applied to the corresponding template μ-map to generate a subject-specific attenuation map for the final reconstruction. A postreconstruction gaussian filter with 1.5-mm full width at half maximum was applied to all reconstructed images.

Two patients with suspected Alzheimer disease underwent amyloid PET scans approximately 110 min after the injection of 300 MBq of [^18^F]florbetaben. The scan time for the first patient, who had been classified as amyloid-negative on the basis of prior whole-body PET/CT scans, was 20 min, whereas that for the second patient with positive amyloid uptake was 10 min. A postreconstruction gaussian filter with a 3.0-mm full width at half maximum was applied to both datasets, which were corrected for attenuation using template-based attenuation correction. The reconstructed images were quantitatively analyzed using BTXBrain software (Brightonix Imaging Inc.), with which SUV ratios (SUVRs) were calculated using the cerebellar cortex as the reference region, based on PET-only segmentation (24).

For dopamine transporter imaging, a 65-y-old woman with suspected Parkinson disease underwent a 20-min *N*-(3-[^18^F]fluoropropyl)-2β-carbomethoxy-3β-(4-iodophenyl)nortropane ([^18^F]FP-CIT) PET scan, which was reconstructed with template-based attenuation correction. The injected dose and uptake time for the patient were 192 MBq and 133 min, respectively. Specific binding ratios (SBRs) for individual striatal subregions were quantitatively assessed using BTXBrain software, with the occipital cortex serving as the reference region (25).

In addition, a 10-min breast PET scan in the prone position was performed on a 63-y-old woman 68 min after the intravenous injection of 381 MBq of [^18^F]FDG. The patient was referred to this study to aid in monitoring recurrence of intraductal carcinoma of the right breast. Contour-based uniform attenuation correction was applied. A gaussian filter with 3.0-mm full width at half maximum was also applied to the dopamine transporter and breast scans.

All human studies were approved by the institutional review board of Seoul National University Hospital, and written informed consent was obtained from all participants.

## RESULTS

### Hoffman Phantom Study

**FIGURE 3.**
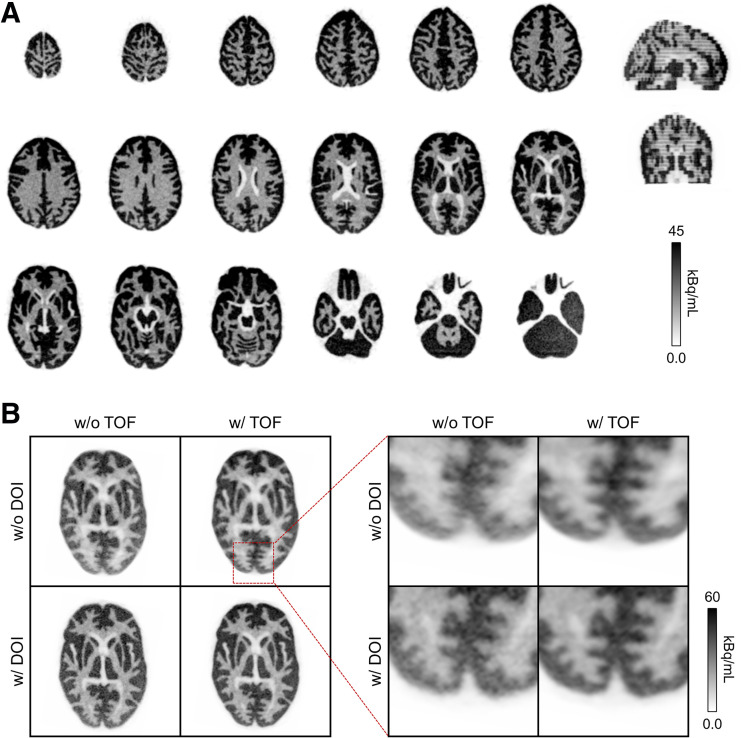
3D Hoffman phantom study. (A) Reconstructed images incorporating both DOI and TOF information. Layered activity patterns are well resolved in coronal and sagittal views. (B) Reconstructed images with 4 different configurations: without DOI and TOF, with DOI only, with TOF only, and with both DOI and TOF. Incorporating DOI reduces radial blurring toward periphery of FOV, whereas TOF improves noise properties and overall image quality. Magnified images of regions within red dashed box are shown in right panel.

The 3D Hoffman brain phantom images reconstructed with DOI and TOF information demonstrated remarkable spatial resolution, with clear visualization of cortical and subcortical structures without noticeable artifacts (Fig. 3A). Quantitatively, the measured gray-to-white matter contrast was 2.16, which is lower than the intended contrast ratio defined in the Hoffman phantom design. The Hoffman phantom was designed with gray matter layers 4 times thicker than the white matter in each slice, yielding an apparent gray-to-white matter contrast close to 4:1 in a low-resolution PET scanner due to partial-volume blurring. However, in high-resolution systems, partial-volume effects are reduced, resulting in measured contrast values that are lower than the intended ratio, consistent with reports from other advanced brain-dedicated PET scanners, such as the NeuroEXPLORER (23). The segmented gray/white matter regions used for quantification are shown in Supplemental Figure 1.

The incorporation of DOI mitigates radial blurring near the periphery of the FOV, whereas TOF further improves noise characteristics, as demonstrated in Figure 3B. The measured SNR_GM_ values were 3.97 (without DOI and without TOF), 5.77 (without DOI and with TOF), 4.21 (with DOI and without TOF), and 6.05 (with DOI and with TOF). Incorporating TOF yielded approximately a 1.45-fold increase in SNR, whereas the inclusion of DOI provided an additional improvement by reducing partial-volume blurring and enhancing signal consistency in the gray matter region.

### Brain Glucose Imaging

**FIGURE 4.**
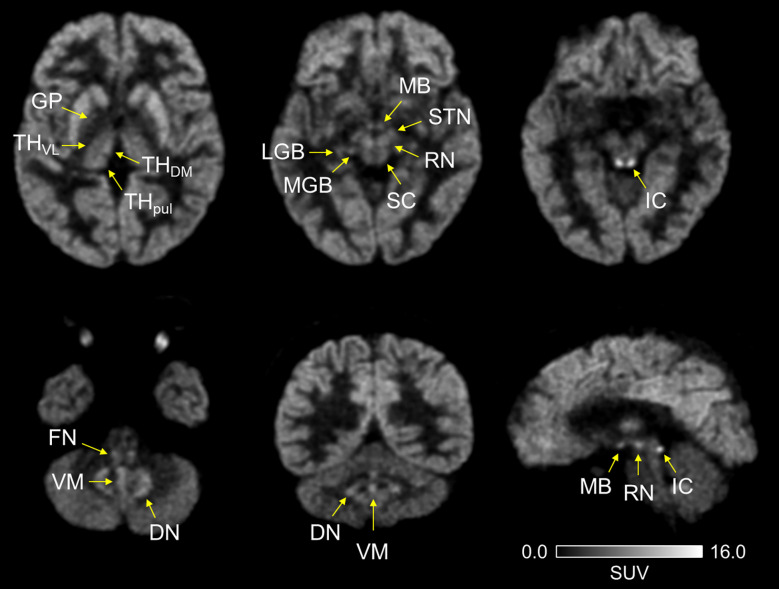
Brain [^18^F]FDG PET in cognitively normal volunteer, demonstrating high-quality images with well-defined cortical uptake and symmetric distribution of glucose metabolism in gray matter, deep cortical structures, and brainstem nuclei. Anatomic structures were visually identified by a nuclear medicine physician. DN = dentate nucleus; FN = fastigial nucleus; GP = globus pallidus; IC = inferior colliculus; LGB = lateral geniculate body; MB = mammillary body; MGB = medial geniculate body; RN = red nucleus; SC = superior colliculus; STN = subthalamic nucleus; TH_DM_ = dorsomedial thalamic nucleus; TH_pul_ = pulvinar nucleus; TH_VL_ = ventrolateral thalamic nucleus; VM = vermis.

In the cognitively normal volunteer, reconstructed images from 30-, 20-, and 10-min scan durations exhibited the expected increase in noise with shorter acquisitions (Supplemental Fig. 2). Despite this, the 20- and 10-min datasets retained acceptable SNRs and preserved sufficient anatomic detail for qualitative assessment. The 30-min dataset (Fig. 4) produced high-quality images with well-defined cortical uptake patterns. Glucose metabolism in the gray matter, deep cortical structures, and nuclei within the midbrain and brainstem was clearly distinguished from that in the subcortical white matter, showing a symmetric distribution. No abnormal hypermetabolic or hypometabolic lesions were detected.

### Amyloid Imaging

**FIGURE 5.**
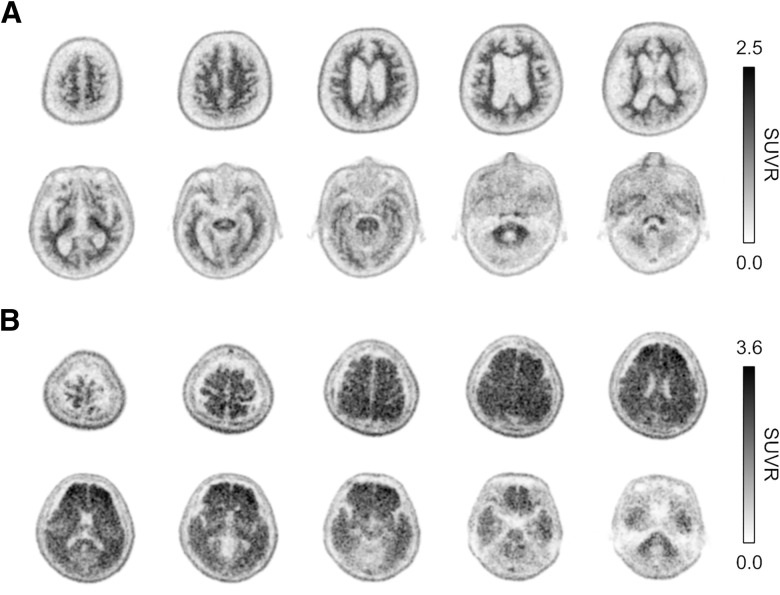
Amyloid PET imaging in patients with suspected Alzheimer disease. (A) Amyloid-negative case with higher [^18^F]florbetaben retention in white matter than gray matter, with preserved spiculated white matter pattern and clear gray–white matter contrast. (B) Amyloid-positive case with diffuse cortical binding in frontal, temporal, and parietal lobes.

The amyloid-negative case shown in Figure 5A demonstrated higher [^18^F]florbetaben retention in the white matter than in the gray matter, preserving the characteristic spiculated pattern of white matter tracts and producing marked gray–white matter contrast. A radiotracer deficit was noted in the bilateral cerebellum, which served as the reference region. Structural changes, such as ventricular enlargement and cortical gray matter atrophy, were also evident. The patient was presumably referred for evaluation of cognitive decline. These imaging findings suggest that the cognitive symptoms are unlikely due to Alzheimer disease. However, the observed cortical atrophy and ventriculomegaly indicate neurodegeneration of a non-Alzheimer origin, such as subcortical vascular dementia or other etiologies.

The amyloid-positive case shown in Figure 5B demonstrated diffuse cortical binding in the frontal, temporal, and parietal lobes, as well as in the posterior cingulate cortex. Elevated [^18^F]florbetaben uptake in the gray matter reduced the contrast with adjacent white matter structures. Relative sparing of the precentral and postcentral cortices and the occipital cortex was observed. Additionally, prominent radiotracer retention was noted in the bilateral caudate nuclei.

Quantitative analysis using BTXBrain further supported these visual interpretations. The amyloid-negative case showed a global SUVR of 1.03, whereas the amyloid-positive case demonstrated a global SUVR of 1.73. These values fall within the reported SUVR ranges for [^18^F]florbetaben, clearly distinguishing amyloid-negative from amyloid-positive patterns as described in prior studies (26).

### Dopamine Transporter Imaging

**FIGURE 6.**
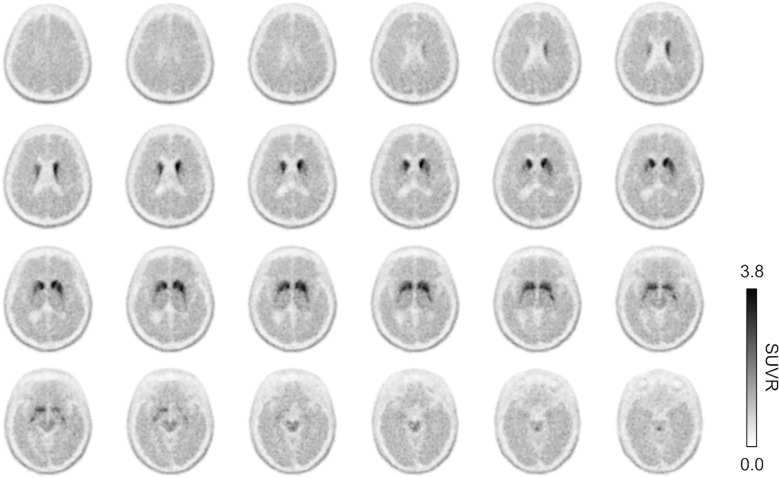
Dopamine transporter imaging with [^18^F]FP-CIT PET on patient with suspected Parkinson disease with reduced tracer binding in bilateral putamen but relative preservation of caudate uptake.

The patient with suspected Parkinson disease demonstrated reduced tracer binding in the putamen bilaterally, with relative preservation of caudate uptake, consistent with early-stage Parkinsonian patterns (Fig. 6).

In line with these findings, regional SBRs demonstrated asymmetry between the left and right striatum. Particularly, the dorsal striatum showed reduced tracer binding on the right side (SBR, 0.78) compared with the left side (SBR, 1.29). A similar pattern was observed in the caudate nucleus (left, 1.79; right, 1.10). Segmental analysis of the putamen revealed a marked decline in the posterior putamen (left, 0.66; right, 0.34), whereas the anterior putamen exhibited relatively preserved uptake (left, 1.49; right, 0.97). The SBRs observed in this case are consistent with the previously reported quantitative ranges for early Parkinson disease (27).

### Breast Glucose Imaging

**FIGURE 7.**
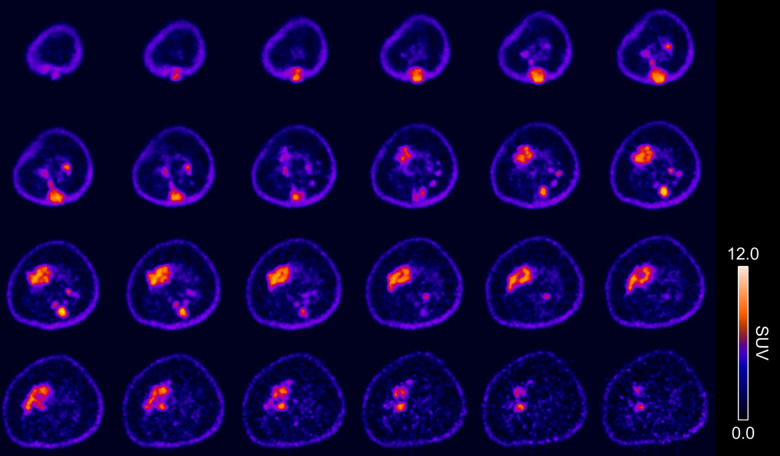
Prone-position [^18^F]FDG PET image of patient with recurrent intraductal carcinoma of right breast. High spatial resolution and target-to-background contrast enable clear lesion delineation and detailed visualization of tumor heterogeneity.

In the patient with recurrent intraductal carcinoma of the right breast, prone-position [^18^F]FDG PET clearly delineated multiple lesions with high target-to-background contrast (Fig. 7). Lesion localization and extent were consistent with prior diagnostic work-up. Multicentric masses and nodules with heterogeneous hypermetabolic activity were observed throughout the right breast. Additionally, diffuse hypermetabolism with associated skin thickening was noted, indicating skin invasion. The image provided excellent contrast between the tumor and the surrounding normal breast tissue, enabling clear lesion delineation and detailed visualization of tumor heterogeneity.

## DISCUSSION

In this study, we have developed the PHAROS, a high-resolution, multifunctional PET imaging system integrating TOF and DOI technologies, and reported the results of its first-in-human evaluation. Featuring a movable detector head and transformable patient couch, PHAROS supports flexible patient positioning for brain, breast, and extremity imaging within a compact footprint. Initial performance assessments using a 3D Hoffman brain phantom and diverse clinical PET applications demonstrated its ultra–high spatial resolution, excellent image contrast, and precise lesion delineation.

Through the implementation of dual-ended readout architecture and advanced front-end electronics (22,28), the PHAROS system achieves sub–4-mm DOI resolution and sub–250-ps coincidence timing resolution. In the Hoffman phantom study, these TOF and DOI capabilities resulted in more than a 1.5-fold improvement in image SNR, increasing from 3.97 without TOF and DOI to 6.05 when both TOF and DOI were incorporated. These results experimentally demonstrate that the combined DOI and TOF performance of PHAROS translates into tangible improvements in image quality for high-resolution brain PET imaging.

The versatility of the PHAROS system makes it suitable for both routine clinical practice and advanced research, particularly in studies requiring flexible patient positioning and high spatial resolution. In the context of neurodegenerative diseases, PHAROS holds promise for enabling earlier and more accurate diagnoses, supporting timely therapeutic interventions. In amyloid PET imaging, even modest improvements in spatial resolution have been shown to significantly reduce partial-volume effects and enhance both cross-sectional and longitudinal quantification of amyloid burden—improving sensitivity to early pathologic changes (e.g., Centiloid separation) (29). Similarly, for dopamine transporter imaging, PET systems with superior spatial resolution and contrast enable more precise delineation of subregional binding patterns and improve quantification of striatal dopamine transporter availability, which is critical for differentiating parkinsonian disorders (30). Its impact on neuroimaging can be further amplified when integrated with automated and objective PET quantification methods, as demonstrated in this study (24,31). Moreover, the application of dynamic imaging protocols may provide novel insights into tracer kinetics and functional brain mapping. Although these approaches still require validation in real-world clinical settings, the high-resolution PHAROS offers a promising platform to explore and translate these emerging applications into clinical practice.

Beyond high-resolution neuroimaging, the PHAROS system can be configured for breast and extremity imaging, allowing detailed visualization of peripheral tumors, vascular abnormalities, and inflammatory musculoskeletal conditions. This flexible configuration underscores the potential of PHAROS to serve as a complementary imaging tool alongside whole-body PET systems in various specialized clinical workflows.

Although the initial results presented in this study are promising, several limitations warrant discussion. The current study involved a limited number of subjects, and further large-scale clinical investigations are necessary to validate diagnostic performance of PHAROS across diverse populations and pathologies. In addition, the limited FOV inherent to the small-ring geometry requires careful consideration in clinical applications. Optimal patient positioning may be challenging in subjects with broad shoulders and short necks during brain imaging, and lesions near the chest wall during breast imaging may lie close to the edge of the FOV. Nevertheless, appropriate positioning strategies and scanning protocols can mitigate many of these limitations. Further studies are needed to refine patient setup guidelines and optimize imaging workflows to fully leverage the high-resolution capabilities of PHAROS in dedicated oncologic applications.

The current system does not yet implement high count-rate correction techniques such as pulse pile-up correction. Incorporating these methods is expected to make the NECR performance more comparable to that of conventional whole-body PET scanner. Moreover, the reconstruction algorithms used did not incorporate point-spread function modeling or motion correction, which could further enhance image quality of this ultra–high-resolution PET scanner. Future software developments could also integrate these advanced reconstruction techniques along with artificial intelligence–based noise reduction and image correction to maximize the clinical utility of PHAROS (32,33).

## CONCLUSION

By combining excellent timing resolution with DOI encoding, the PHAROS system delivers high-quality phantom and human PET images, demonstrating clear potential for accurate diagnosis of brain, breast, and other diseases. Its innovative design and advanced imaging capabilities offer promise for enhancing diagnostic precision and facilitating research into various human diseases.

## DISCLOSURE

This work was supported by the Korea Medical Device Development Fund grant funded by the Korea government (the Ministry of Science and ICT, the Ministry of Trade, Industry and Energy, the Ministry of Health & Welfare, the Ministry of Food and Drug Safety) (project number: 1711137868, RS-2020-KD000006). Guen Bae Ko, Kyeong Yun Kim, Seung Kwan Kang, Jaehun Lee, Raehun Jung, Dong Jin Kwak, Jeong-Whan Son, Seong A Shin, and Jae Sung Lee are employees of Brightonix Imaging Inc. No other potential conflict of interest relevant to this article was reported.

## Notes

KEY POINTS **QUESTION:** Can a newly developed, high-resolution TOF/DOI PET system (PHAROS) provide clinically useful brain and breast imaging in various patient positions, and how does it perform in its first-in-human studies? **PERTINENT FINDINGS:** The PHAROS produced ultra–high-resolution PET images with excellent contrast and anatomical detail across multiple tracers and applications, including [^18^F]FDG brain PET, amyloid and dopamine transporter imaging, and prone-position breast PET. **IMPLICATIONS FOR PATIENT CARE:** PHAROS’s versatility and image quality may enable more accurate diagnosis of neurologic and oncologic diseases, improved lesion characterization, and enhanced patient comfort through tailored positioning.
